# Highly Enhanced Mechanical, Thermal, and Crystallization Performance of PLA/PBS Composite by Glass Fiber Coupling Agent Modification

**DOI:** 10.3390/polym15153164

**Published:** 2023-07-26

**Authors:** Zhiqiang Fan, Junchang Gao, Yadong Wu, Dewu Yin, Shunxing Chen, Hua Tu, Tiantian Wei, Chaoran Zhang, Haoxiang Zhu, Huile Jin

**Affiliations:** 1 Key Laboratory of Leather of Zhejiang Province, College of Chemistry and Materials Engineering, Wenzhou University, Wenzhou 325035, China; fanzhiqiang1015@163.com (Z.F.); huilejin@wzu.edu.cn (H.J.); 2 Cangnan Research Institute, Wenzhou University, Wenzhou 325035, China; 3 Institute of New Materials and Industrial Technology, Wenzhou University, Wenzhou 325035, China

**Keywords:** polylactic acid (PLA), polybutylene succinate (PBS), glass fiber (GF), composite material modification, crystallinity

## Abstract

To improve the toughness and heat resistance of polylactic acid (PLA), polybutylene succinate (PBS) was sufficiently blended with PLA as the base matrix, and the glass fiber (GF) that was modified with 3-aminopropyltriethoxysilane (KF-GF) was added as the reinforcement. The results demonstrated a noteworthy boost in both mechanical and heat resistance properties when employing KH-GF, in comparison to pristine GF. When the content of KH-GF reached 20%, the tensile, flexural, and IZOD impact strength of the composites were 65.53 MPa, 83.43 MPa, and 7.45 kJ/m^2^, respectively, which were improved by 123%, 107%, and 189% compared to the base matrix, respectively. This enhancement was primarily attributed to the stronger interfacial adhesion between KH-GF and the PLA/PBS matrix. Furthermore, the Vicat softening temperature of the composites reached 128.7 °C, which was a result of increased crystallinity. In summary, the incorporation of KH-GF into PLA/PBS composites resulted in notable enhancements in their mechanical properties, crystallinity, and thermal characteristics. The high performance KH-GF-reinforced PLA/PBS composite showed a broad application potential in the field of biodegradable packaging, biodegradable textiles, and biodegradable plastic bags.

## 1. Introduction

In recent years, with the development and progress of society, there has been an increasing emphasis on the development and utilization of environmentally friendly materials. Biodegradable polymers have become a focal point for numerous researchers in this field. As non-renewable fossil fuels and petroleum resources become depleted, industries worldwide are seeking renewable resources to meet the demands of consumers and keep up with the times. Therefore, the development and modification of green polymers and biodegradable materials have become an inevitable trend in today’s society [1,2,3]. Polylactic acid (PLA) has gained favor among researchers and is considered the most competitive biodegradable biomaterial. It has been approved as a biodegradable medical material not only in the United States but also in other countries [4,5,6,7]. PLA, a linear thermoplastic aliphatic polyester, was first discovered by American polymer chemist Carothers and possesses excellent biodegradability and biocompatibility [8,9,10,11,12]. Additionally, PLA exhibits high strength, high modulus, and good processability, making it an important substitute for petroleum-based general-purpose plastics such as polypropylene (PP) and polystyrene (PS) [13,14,15]. However, the inherent brittleness and poor heat resistance of PLA limit its applications and development. Specifically, PLA lacks flexibility, has poor impact resistance, and exhibits a low Vicat softening temperature. Hence, it is essential to improve the toughness and heat resistance of PLA, which is crucial for expanding its application areas.

Currently, the most economically practical method for modifying PLA is physical blending. Many fully biodegradable materials have gained the trust of researchers, including polyhydroxyalkanoates (PHA), polycaprolactone (PCL), polyethylene oxide (PEO), polybutylene succinate (PBS), and polybutylene adipate-co-terephthalate (PBAT) [16,17,18]. Among these biodegradable materials, PBS exhibits excellent mechanical properties and heat resistance; however, PBS materials also have certain limitations, such as softness, low gas barrier, and high cost [19]. Aliotta, L et al. [20] reviewed the synthesis, production, thermal, morphological and mechanical properties, and biodegradability of PBS and its main copolymers, and introduced their main applications. This work will expand the research of PBS to a large extent, and has great research significance for PLA/PBS composites. Incorporating PBS into PLA can effectively enhance the thermal and mechanical properties of the PLA composite. Previous studies have shown that the addition of PBS improves the thermal performance of PLA, with better heat resistance observed at higher PBS contents [21,22,23,24]. Su et al. [25] reported the research progress on improving the properties of PLA/PBS blends in recent years, and reviewed the modification methods of PLA/PBS, such as simple blending, plasticizing, reaction compatibilization, and copolymerization, so as to improve the toughness and heat resistance of PLA/PBS blends. The simple blending of PLA and PBS will increase the elongation at break, but decrease the tensile strength and modulus. This has greatly expanded the research methods of PLA/PBS composites. There are few studies on the plasticizing effect of PLA/PBS. In general, the use of plasticizers reduces the intermolecular force and improves the fluidity of polymer chains, thus improving the processability and flexibility [26]. Compared with plasticizing modification, a lot of research has been conducted on reaction capacity enhancement. This method can effectively improve the toughness of PLA/PBS composites. Among the different reactivity compatibilizers, DCP showed the highest efficiency. Wang et al. [27] reported that compression-molded sheets of a PLA/PBS (80/20) blend with DCP had the same elongation at break (250%) as the ones without DCP, while the tensile modulus and strengths decreased, but the notched Izod impact strength increased about eight times. Compared with reactive capacity enhancement, copolymerization as a non-reactive capacity enhancement method can achieve a moderate improvement in elongation at break. In addition, copolymers have been shown to plasticize and nucleate PLA/PBS blends. The synergistic effect on performance improvement is achieved by combining linear block copolymers and branched copolymers [28,29].

It is well known that the slow crystallization rate of PLA leading to low crystallinity is the main cause of its poor heat resistance. The addition of nucleating agents is a simple and efficient method to increase the crystallization rate and improve the crystallinity of polymers [30,31,32]. Among various nucleating agents, fibers have distinct advantages due to their ability to promote polymer crystallization and significantly strengthen the matrix of the polymer, resulting in composite materials with excellent properties. Glass fiber (GF) is the most commonly used fiber nucleating agent due to its low cost, uniform surface structure, high strength, and good heat resistance [33,34,35,36,37]. Wang et al. [38] conducted research on GF-reinforced PLA composites and obtained objective data. The study showed that when the GF content reached 20 wt%, the crystallinity increased to 33.48%. Furthermore, they achieved further improvements in crystallinity through a heat treatment process. The increasing intensity of XRD diffraction peaks clearly indicated an increase in crystallinity with an increasing GF content. Importantly, the content, length, and interfacial bonding capacity of GF are crucial factors affecting the mechanical properties of composite materials. Yin et al. [39] investigated the effects of modified GF on the mechanical properties and crystallinity of PLA composites. They first modified the GF surface with graphene oxide (GO) and then treated it with 3-aminopropyltriethoxysilane (APTES). The crystallinity of the resulting PLA-GF/CRG composite material increased from 27.61% to 51.29%. Additionally, when the GF content was 10 wt%, the tensile strength of the PLA-GF/CRG composite material increased by approximately 63% compared to pure PLA.

Therefore, to develop a biodegradable composite material with high strength, toughness, and good heat resistance, this study developed a modified glass fiber, KH-GF, as a reinforcement. Based on a PLA/PBS binary blend, a highly heat-resistant and tough biodegradable composite material was prepared. Furthermore, GF was added, and its surface was modified to investigate the mechanical and thermal properties of PLA/PBS/GF composites at different ratios. The practical application of the PLA/PBS composite in biodegradable materials is expanded, so that it can have better durability and practicability.

## 2. Materials and Methods

### 2.1. Materials and Sample Preparation

Polylactic acid (PLA-201) was provided by Zhejiang Haizheng Biotechnology Co., Ltd. (Taizhou, China). Polybutylene succinate (PBS-803S) was provided by Xinjiang Lanshan Tuniu Corporation (Changji, China). Glass fiber (GF, 3 mm, Grade A) was obtained from Shuolong Mineral Products Co., Ltd. (Hengshui, China). The chain extender ADR4468 (containing epoxy functional groups) was provided by BASF Ludwigshafen GmbH (Ludwigshafen, Germany). Calcium carbonate (CaCO_3_) was supplied by Qingdao Zhongxin Huamei Plastics Co., Ltd. (Qingdao, China). The 3-aminopropyltriethoxysilane (KH-550) was provided by Beijing Huaweiruike Chemical Technology Co., Ltd. (Beijing, China).

### 2.2. Preparation of KH-GF

**Figure 1:**
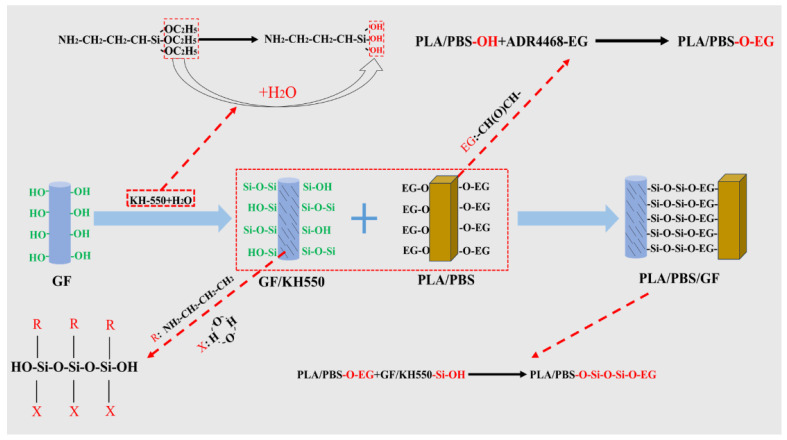
Mechanism diagram of the modified composite material.

Firstly, glass fiber was completely immersed in a KH-550 aqueous solution (mass ratio of 1:80) for 6 h. The solution was then filtered, and the glass fiber was placed in a well-ventilated environment to air dry naturally. The prepared modified glass fiber was referred to as KH-GF. Figure 1 presents a mechanism diagram of the modified composite material. The mechanism can be explained as follows: firstly, the silane coupling agent KH-550 undergoes hydrolysis to form silanol (Si-OH) molecules. These silanol molecules react with the -OH groups on the surface of the glass fiber, forming covalent bonds of silicon-oxygen-silicon (Si-O-Si) [40,41]. This attaches the silane coupling agent molecules to the surface of the glass fiber and forms a protective layer, preventing damage to the glass fiber due to environmental factors. This achieves the bonding of the silane coupling agent with the glass fiber surface. Secondly, during the reaction, the substance containing epoxy functional groups (ADR4468) undergoes ring-opening reactions, generating compounds with reactive functional groups (usually epoxy functional groups). These compounds undergo esterification reactions with the -OH groups on the surface of PLA/PBS, forming new ester linkages. Finally, the PLA/PBS with hydroxyl functional groups reacts with the compound containing Si-OH chemical bonds (GF modified by the silane coupling agent), forming new siloxane bonds, thereby tightly binding the materials together.

### 2.3. Preparation of Modified PLA/PBS Composite Materials

**Figure 2:**
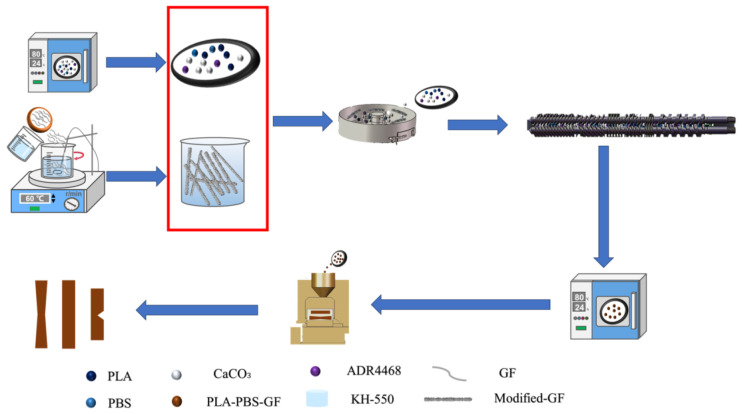
Flowchart of the preparation process of the modified composite material.

**Figure 3:**
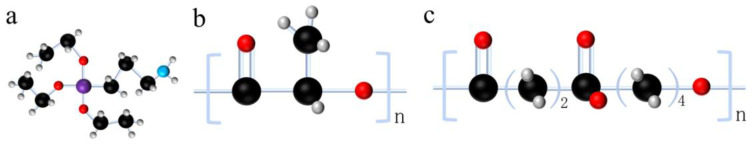
(**a**) Chemical structure of the coupling agent KH550, (**b**) Chemical structure of PLA, (**c**) Chemical structure of PBS. (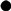 = C; 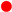 = O; 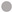 = H; 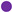 = Si; 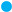 = N).

PLA, PBS, glass fiber, and calcium carbonate were placed in a vacuum oven and dried at 80 °C for 24 h to remove the moisture. The dried materials were uniformly mixed in a certain ratio to form the base matrix using a mixer. Then, additives were added according to Table 1, and thorough mixing was performed in a high-speed mixer (mixing time: 10 min, speed: 300 rpm). The mixture was then processed using a co-rotating twin-screw extruder (TSE-35, Nanjing Dali Extrusion Machinery Co., Ltd., Nanjing, China) at temperatures ranging from 130 °C to 190 °C, with a main screw speed of 360 rpm and a feed rate of 6 rpm. The obtained pellets were dried in a vacuum oven at 80 °C for 24 h. Finally, injection molding was performed using an injection molding machine (TY-200, Dayu Machinery Co., Ltd., Zhengzhou, China). Processing temperature: 170 °C, 175 °C, 180 °C, mold temperature: 30 °C, injection pressure: 125 bar, holding pressure: 50 bar, cooling time: 30 s. The dimensions of the resulting specimens for tensile testing were 160 mm × 10 mm × 4 mm. The dimensions of the specimens for bending tests were 150 mm × 10 mm × 4 mm. The dimensions of the notched impact test specimens were 80 mm × 10 mm × 4 mm. In addition, Figure 2 presents the specific preparation process of the composite material, and Figure 3 shows the chemical structures of some of the materials.

### 2.4. Testing Methods

#### 2.4.1. Fourier Transform Infrared Spectroscopy (FTIR)

The possible functional groups formed between the components of the PLA/PBS composite material were studied using Fourier Transform Infrared Spectroscopy (FTIR). The infrared spectrometer of Perkin Elmer, UK. The mode was ATR, the sample thickness was 1 mm, and the model was L1230104, in the range of 400–4000 cm^−1^, with a resolution of 4 cm^−1^ and 8 scans.

#### 2.4.2. Mechanical Properties

Mechanical properties of the samples were tested using a servo-controlled tensile testing machine (Model GT-AI7000-L10, Dongguan Gaotie Detection Instrument Co., Ltd., Dongguan, China). Tensile properties and flexural properties were tested according to GB1040 and GB1042, respectively, at speeds of 10 mm/min and 20 mm/min, and the load was 10,000 kgf. The notched impact strength was tested using an impact testing machine (Model JBS-3002, Jinan Liangong Testing Technology Co., Ltd., Jinan, China) following the standard GB/T1843-2008, and the value of the pendulum was 3.46 V/2.75 J. The tests for tensile and impact properties were repeated ten times at room temperature, and the average values were calculated.

#### 2.4.3. Thermal Properties

The thermal stability of the samples was analyzed using a thermogravimetric analyzer (Diamond TG-DTA/Spectrum GX, PerkinElmer, Waltham, MA, USA). A 5–10 mg sample was sealed in a ceramic crucible and heated from 40 °C to 600 °C at a rate of 10 °C/min under a nitrogen environment. The gas flow rate was 200 mL/min.

The Vicat softening temperature (VST) of the samples was measured using a computerized heat deformation temperature tester (Suzhou Yano Industry Co., Ltd., Suzhou, China). The measurement was performed in a silicone oil bath at a 5 °C/min heating rate. Rectangular specimens were subjected to a constant load of 10 N, and the VST was determined when the needle penetrated the sample to a depth of 1 mm. The average value was calculated based on five repeated tests.

#### 2.4.4. Crystallization Properties

Crystallization analysis of the samples was conducted using a differential scanning calorimeter (DSC) Q1000 from TA Instruments (New Castle, DE, USA). A total of 5–10 mg of sample was sealed in an aluminum pan and heated–cooled–heated in a nitrogen environment at a rate of 10 °C/min within the temperature range of 40 °C to 200 °C. The gas flow rate was 50 mL/min. An isothermal hold was performed for 10 min after the first heating to eliminate any thermal history effects. The DSC data were analyzed using the universal analysis program provided by TA Instruments. The crystallization temperature (Tc), cold crystallization temperature (Tcc), melting temperature (Tm), crystallization enthalpy (∆Hc), melting enthalpy (∆Hm), and cold crystallization enthalpy (∆Hcc) of the composite material were calculated according to Formula (1) to determine the crystallinity χ:

(1) $$\chi =\frac{∆Hm-∆Hcc}{{∆H}_{m0}}\times 100\%$$

where ∆Hm_0_ is the melting enthalpy of pure PLA estimated as 93.6 J/g [42], ∆Hm is the melting enthalpy of the composite material, and ∆Hcc represents the cold crystallization enthalpy of the composite material.

#### 2.4.5. Rheological Properties

The flowability of the samples was analyzed using a melt flow index tester (model GT-7100-MI, Dongguan Gaotie Detection Instrument Co., Ltd., Dongguan, China). The test was conducted at a temperature of 190 °C with a 2.16 kg weight. The material was loaded for 15 s. The melt flow rate (MFR) of the composite material was calculated according to Formula (2):

(2) $$MFR=\frac{m÷n}{t}\times 600(g/10\;min)$$

where *m* refers to the total mass of the extruded sample, n is the number of samples extruded, and *t* represents the loading time in seconds.

#### 2.4.6. Microscopic Morphology

After the impact test, the fracture surface morphology of the samples was observed using a scanning electron microscope (Nano SEM200, FEI Company, Hillsboro, OR, USA). Prior to SEM observation, the samples were sputter-coated for gold deposition. In the test, the chamber pressure was less than 6 × 10^−3^ Pa, and the test voltage was 10 KeV.

## 3. Results and Discussion

### 3.1. FTIR Spectroscopy

**Figure 4:**
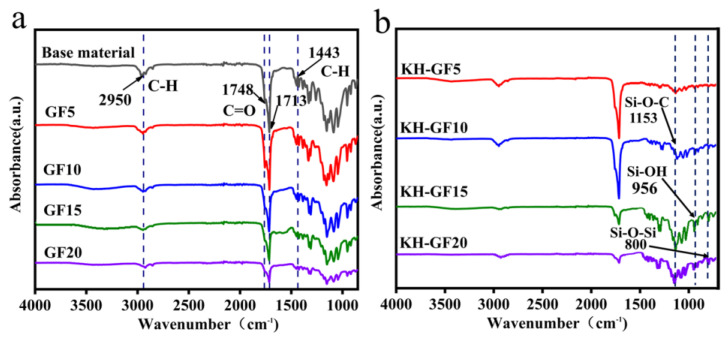
FTIR spectra of modified PLA/PBS-based composites with different additions of GF and KH-GF: (**a**) GF, (**b**) KH-GF.

Figure 4 shows the FTIR spectra of modified PLA/PBS-based composites with different additions of GF and KH-GF. By comparing the infrared absorption peaks before and after modification, it can be observed that the peak positions do not exhibit significant shifts. However, significant changes in peak intensity are observed in the range of 1000–1700 cm^−1^ as the content of glass fiber varies, with an increasing trend corresponding to the increase in glass fiber content. Compared to unmodified GF, the GF modified with the coupling agent exhibits higher absorption intensity at the stretching vibration peak of C=O at 1713 cm^−1^. This is mainly attributed to the enhanced dispersibility resulting from the increased content of GF, which strengthens the effect of the coupling agent, leading to a stronger absorption peak. Additionally, the peak at 1443 cm^−1^ can be attributed to the deformation of C-H in CH_3_, while the peak at 1153 cm^−1^ corresponds to the stretching of asymmetric C-O in Si-O-C. These peaks arise from the -COOH and -C=O end groups of PLA and the -C=O component of PBS [43]. Furthermore, in the FTIR spectra of the modified GF, new peaks appear at 956 cm^−1^ and 800 cm^−1^, and their intensities become more pronounced as the GF content increases. This could be due to the interaction between the coupling agent and the PLA/PBS matrix, forming Si-OH and Si-O-Si bonds with the -OH groups, thereby enhancing the interfacial strength between GF and the matrix, and improving the intermolecular bonding at the interface. The chemical properties of the obtained PLA/PBS composites are more polar than other matrix materials such as PP, which will affect the interface adhesion properties with polar fillers [44], evidenced by the force spectroscopy measurements [45].

### 3.2. Mechanical Properties

#### 3.2.1. Tensile Properties

**Figure 5:**
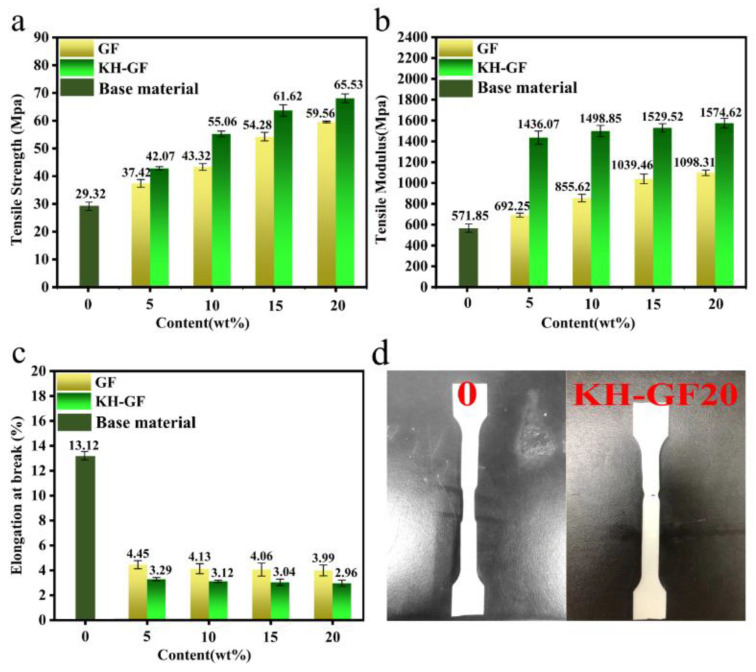
Tensile properties of modified composite materials with different additions of GF and KH-GF: (**a**) Tensile strength, (**b**) Tensile modulus, (**c**) Elongation at break, (**d**) Schematic diagram of tensile specimen.

Figure 5 presents the tensile strength and tensile modulus of GF and KH-GF-reinforced composite materials. As shown in Figure 5a, the tensile strength of the composite material increases with the addition of GF and KH-GF. Compared to the GF-reinforced composite material, the enhancement in tensile performance is more significant in the case of KH-GF. When the KH-GF content reaches 20%, the composite material exhibits a tensile strength of 65.53 MPa, which is a 123% improvement compared to GF0. Similarly, as shown in Figure 5b, the tensile modulus follows a similar trend. However, as shown in Figure 5c, the fracture elongation of the modified composite material exhibits an opposite trend. The addition of GF reduces the fracture elongation to around 4% when 5% of GF is added, while the KH-GF-modified composite material shows even lower fracture elongation. When the GF content is 20%, fracture elongation is reduced by approximately 77%. These results indicate that KH-GF enhances the strength and modulus of the PLA/PBS composite material but sacrifices the fracture elongation to some extent. Additionally, Figure 5d presents schematic diagrams of the tensile samples before and after modification. From this figure, we can visually observe the fracture behavior of the samples. This can be attributed to the fact that GF itself has good tensile properties, and after modification with the coupling agent, the interfacial bonding strength between KH-GF and the PLA/PBS blend is enhanced, resulting in a significant increase in tensile strength. However, with the increased content of glass fiber, a large amount of short-cut glass fiber hinders the movement of PLA and PBS molecular chains in the plastic matrix, leading to an increase in the elastic modulus and a decrease in fracture elongation. This will potentially limit the applications of the modified composite material to some extent.

#### 3.2.2. Flexural Properties

**Figure 6:**
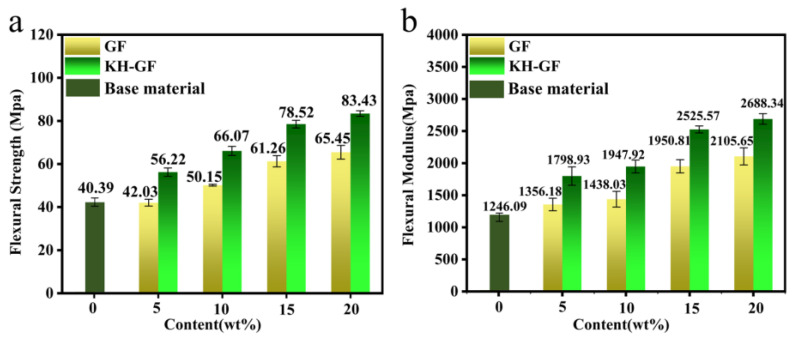
Flexural properties of modified composite materials with different additions of GF and KH-GF: (**a**) Flexural strength, (**b**) Flexural modulus.

Figure 6 presents the flexural strength and flexural modulus of GF/KH-GF-reinforced composite materials. As shown in Figure 6a, the flexural strength of the composite material increases with the addition of GF and KH-GF. Similarly, in Figure 6b, the flexural modulus exhibits a similar trend. When the KH-GF content reaches 20%, the composite material achieves the maximum flexural strength of 83.43 MPa and flexural modulus of 2688.34 MPa. This corresponds to a 27% increase in flexural strength and a 28% increase in flexural modulus compared to the GF-reinforced material. This enhancement can be attributed to the fact that after treatment with the coupling agent, KH-GF further strengthens the interfacial bonding between KH-GF and the PLA/PBS blend, leading to a significant improvement in flexural strength and flexural modulus of the composite material.

#### 3.2.3. Impact Properties

**Figure 7:**
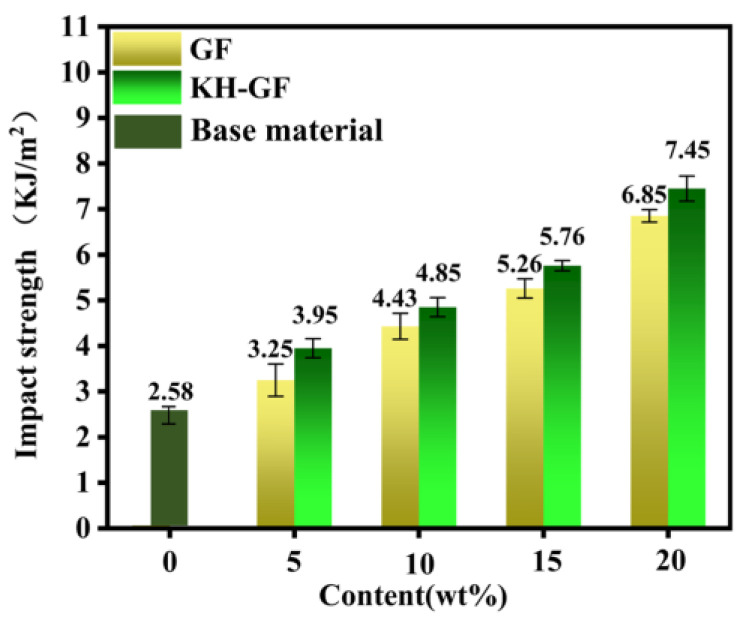
Impact strength of modified composite materials with different additions of GF and KH-GF.

Figure 7 presents the notch Izod impact strength of GF/KH-GF-reinforced composite materials. The results show that the impact strength of the composites is significantly improved by the presence of GF/KH-GF and increases with the increase in its content. It can be observed that the impact strength is only 2.58 KJ/m^2^ when no GF is added. For GF-reinforced composites, when the content of GF reaches 20%, the impact strength is 6.85 KJ/m^2^. When the content of KH-GF is 20%, the impact strength of the composite is higher, reaching 7.45 KJ/m^2^. The increase in impact strength can be explained as the coupling agent can chemically react with the hydroxyl group on the surface of the glass fiber to form a coupling layer, which changes the properties of the glass fiber surface. This coupling layer can provide better fiber surface wettability and cohesiveness, and help to improve the interface bonding force between the glass fiber and the composite material, so that the interface bonding force between the glass fiber and the composite material is enhanced, and the enhancement of the interface bonding force will inevitably increase the energy consumption of the glass fiber drawing process, resulting in the improvement of the impact strength.

### 3.3. Thermal Analysis

#### 3.3.1. DSC Analysis

**Figure 8:**
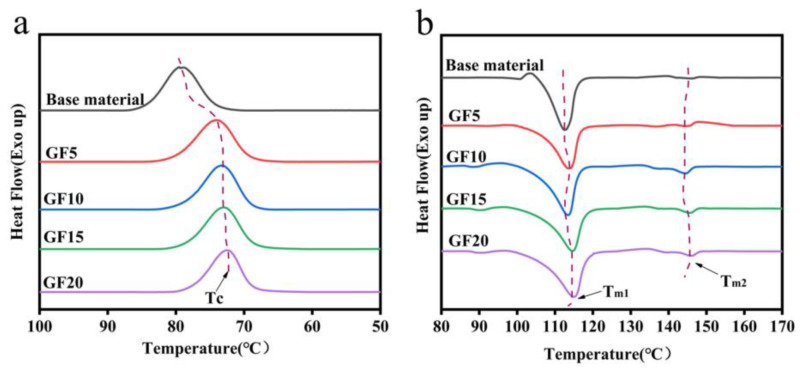
DSC curves of modified composite materials with different additions of GF: (**a**) GF cooling curve, (**b**) GF melting curve.

**Figure 9:**
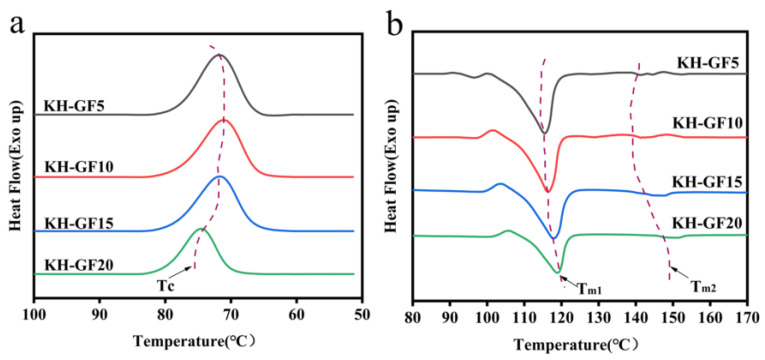
DSC curves of modified composite materials with different additions of KH-GF: (**a**) KH-GF cooling curve, (**b**) KH-GF melting curve.

Further analysis of the thermal properties of the composite materials was conducted. The crystallinity of a material is a crucial factor in improving its heat resistance. As shown in Figure 8a,b and Figure 9a,b and Table 2, the melting curves and crystallization curves of the composite materials were analyzed using DSC, providing information on the crystal temperature (T_C_), crystallization enthalpy (∆H_C_), cold crystallization temperature (T_CC_), cold crystallization enthalpy (∆H_CC_), melting temperature (Tm), and melting enthalpy (∆Hm) of the composite materials, as shown in Table 2. The crystallinity (χ) of the composite materials was calculated according to Formula (1).

As shown in Figure 8 and Figure 9 and Table 2, for the composite material without GF, the T_C_ is 79.53 °C, T_CC_ is 103.26 °C, and the crystallinity is only 34.27%. With the addition of GF, both T_C_ and T_CC_ decrease, indicating a left shift of the crystallization peak. A lower crystallization temperature is more favorable for nucleation and can improve the crystallinity of the composite material to some extent. When the GF content reaches 10%, the crystallinity reaches 41.39%, which is a 20.8% improvement compared to the material without GF. Clearly, the addition of GF effectively accelerates the crystallinity of the composite material. This is because a small amount of GF enlarges the space between molecular chains, increases the nucleation sites between molecules, and plays a prominent role in promoting polymer crystallization. However, when the GF content exceeds 10%, there is a slight decrease in crystallinity. This can be attributed to two reasons. Firstly, with the increase in GF content, agglomeration occurs within the matrix, leading to poor bonding between the matrix and GF, resulting in vacant nucleation sites and a decrease in crystalline performance. Secondly, as the GF content increases, the movement of polymer chains is restricted, preventing the formation of nucleation sites and increasing the hindrance between molecular chains, resulting in a decrease in crystallinity. In comparison to GF, KH-GF exhibits better crystalline performance. When the GF content is 5%, the crystallinity of KH-GF reaches 41.28%. This is because the addition of the coupling agent increases the interfacial adhesion between GF and the PLA/PBS blend, leading to an increase in nucleation sites and thus an increase in crystallinity. Additionally, T_C_ decreases from 79.53 °C to 71.78 °C, and T_CC_ decreases from 103.26 °C to 98.64 °C when KH-GF is added. As is well known, a lower crystallization temperature allows the material to achieve crystallization at a lower temperature. When the crystallization temperature decreases, the thermal motion of molecules weakens, making it easier to form an ordered crystalline structure and consequently enhancing the crystallinity of the composite material. However, with the increase in GF content, the crystallinity decreases. When the content reaches 20%, the crystallinity is only 39.53%. This is because the presence of excessive GF leads to agglomeration, which prevents the expected improvement in crystalline performance. Furthermore, changes in melting points can also be observed. T_m1_ and T_m2_ represent the melting points of PBS and PLA, respectively. From Table 2, we can observe that for GF, T_m1_ remains around 113 °C with increasing GF content, while T_m2_ shows a linear increase with the GF content. This can be explained by the fact that the addition of GF can promote enhanced effects within the matrix, facilitating interactions between PLA/PBS molecules and leading to an increase in the melting point, particularly observed in the increase of PLA’s melting point.

For the addition of KH-GF, both T_m1_ and T_m2_ increase. This is mainly due to the silane coupling agent forming a hydrophilic coating on the surface of GF, enhancing the interaction between GF and PLA/PBS, promoting molecular binding, and further increasing the melting point of the composite material.

Importantly, it can also be observed from Table 2 that with the increase in GF content, the melting enthalpy of GF and KH-GF presents a nonlinear relationship, which is mainly explained by the fact that when a small amount of GF is contained, the dispersion of GF in the matrix is relatively uniform, and the interaction force between GF and the matrix molecules is enhanced, resulting in the increase in melting enthalpy. With the increasing of GF content, the disorder of crystal structure may increase, and this disordered structure may lead to the weakening of the molecular interaction of the matrix, thus reducing the enthalpy of melting.

#### 3.3.2. XRD Analysis

**Figure 10:**
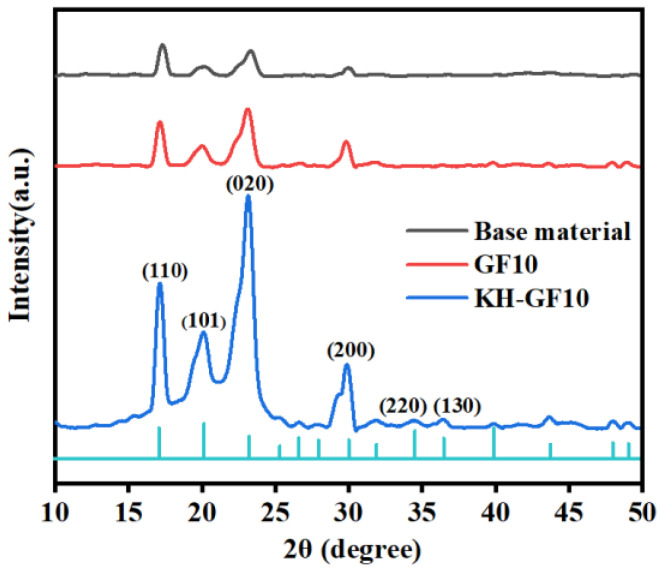
XRD curves of base material, GF10, and KH-GF10.

The samples were further analyzed by XRD. As shown in Figure 10, XRD patterns of the matrix material, GF10, and KH-GF10 are respectively shown. Compared with the matrix material, GF10 and KH-GF10 show stronger diffraction peaks, which is mainly attributed to the improvement of crystallinity, and of course, DSC analysis of the sample can also prove this. It can also be observed from Figure 10 that the diffraction peaks of PLA and PBS appear near 2θ = 17.06°, 20.12°, 23.21°, and 25.32°, corresponding to (110), (101), (020), and (200), respectively, which are consistent with the α crystal patterns of PLA and PBS [38]. Compared with the matrix material and GF10, we can observe that KH-GF10 displays a new diffraction peak at 2θ = 34.53° and 34.46°, indicating that the glass fiber modified by the coupling agent can further improve the crystallinity of the composite material, which is also confirmed by the results of DSC.

#### 3.3.3. TGA Analysis

**Figure 11:**
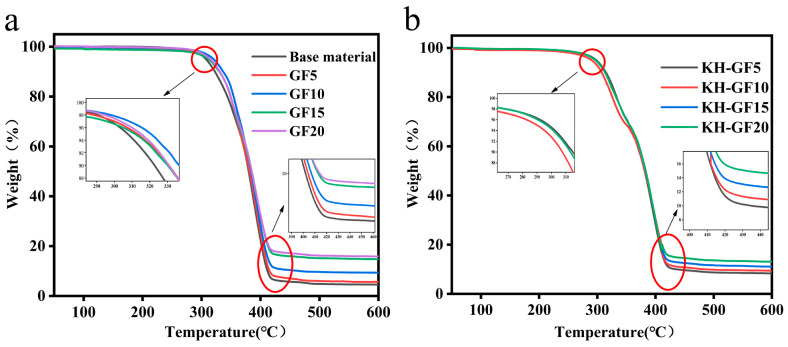
TGA curves of modified composite materials with different additions of GF and KH-GF: (**a**) GF, (**b**) KH-GF.

The thermal stability of PLA/PBS/GF composite materials was further analyzed using TGA characterization. As shown in Figure 11 and Table 3, the TGA curves and decomposition temperatures at various stages are provided for modified composite materials with different additions of GF and KH-GF. From Figure 11, it can be roughly observed that the weight loss rate increases with the increase in GF content, indicating that different proportions of GF can affect the thermal stability of the composite material. Table 3 provides more detailed data, where T_0_, T_5%_, T_10%_, T_50%_, and T_d_ represent the decomposition temperatures when the initial weight loss rates are 0%, 5%, 10%, 50%, and 100%, respectively.

From Table 3, it can be seen that the composite material without GF has an initial decomposition temperature of only 214 °C. However, when the GF content reaches 20%, this value increases to 273 °C. Additionally, as the temperature increases, the decomposition temperatures at various stages also increase with the increase in GF content. Especially when the temperature exceeds 600 °C, the residual mass of the composite material increases from 4.56% to 15.82%. The increase in residual mass percentage indicates better thermal stability of the material. This is also supported by the analysis of crystallinity from the DSC data. Furthermore, it is evident that the addition of the coupling agent significantly improves the thermal stability of KH-GF composite materials. The decomposition temperature at 50% weight loss increases from 379 °C to 389 °C when the coupling agent is added. Moreover, it can be observed that with the increase in KH-GF content, the decomposition temperatures at various stages do not increase but rather show a slight decrease. This can be attributed to the excessive KH-GF narrowing the molecular spaces, hindering the movement of molecular chains, and resulting in a decrease in thermal decomposition temperature. However, compared to GF without coupling agent treatment, KH-GF exhibits better interfacial interaction with the PLA/PBS matrix, leading to a more stable internal structure and improved thermal stability.

#### 3.3.4. Vicat Softening Temperature (VST) Analysis

**Figure 12:**
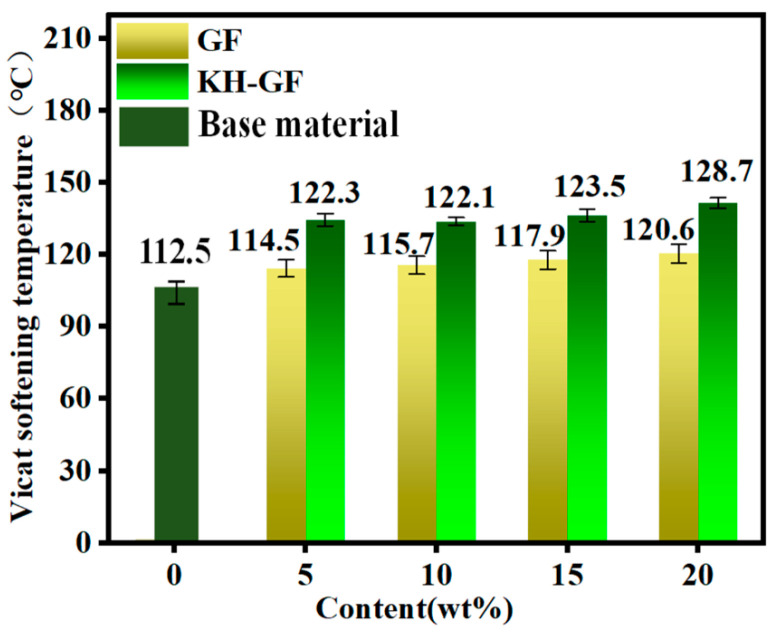
Vicat softening temperature (VST) of modified composite materials with different additions of GF and KH-GF.

In order to provide a more intuitive representation of the heat resistance of PLA/PBS composite materials, the Vicat softening temperature (VST) of injection-molded samples was measured. As shown in Figure 12, the VST of the reference sample is approximately 110 °C, while pure PLA has a VST of around 60 °C [46], indicating an improvement in heat resistance to some extent. This can be explained by the addition of inorganic filler CaCO_3_. On the one hand, CaCO_3_ is a good heat absorber, which can absorb and convert the heat in the composite material into its own heat energy, thereby improving the heat resistance. On the other hand, the addition of CaCO_3_ can improve the interface binding force between the matrix and GF in the composite material, so as to improve the heat resistance of the material.

Furthermore, with the addition of GF, the VST of the composite material also increases, mainly due to the improvement in crystallinity. However, as the GF content increases, the increase in VST becomes marginal, suggesting that the increase in GF content has a negligible effect on the VST of the PLA/PBS composite material. Nevertheless, both GF-reinforced and KH-GF-reinforced composite materials show some improvement in VST with increasing GF content. When the GF content reaches 20%, the VST of the KH-GF composite material increases to around 128 °C. This increase can be attributed to the enhancement in crystallinity, resulting in a higher VST for the KH-GF composite material.

### 3.4. Rheological Properties

**Figure 13:**
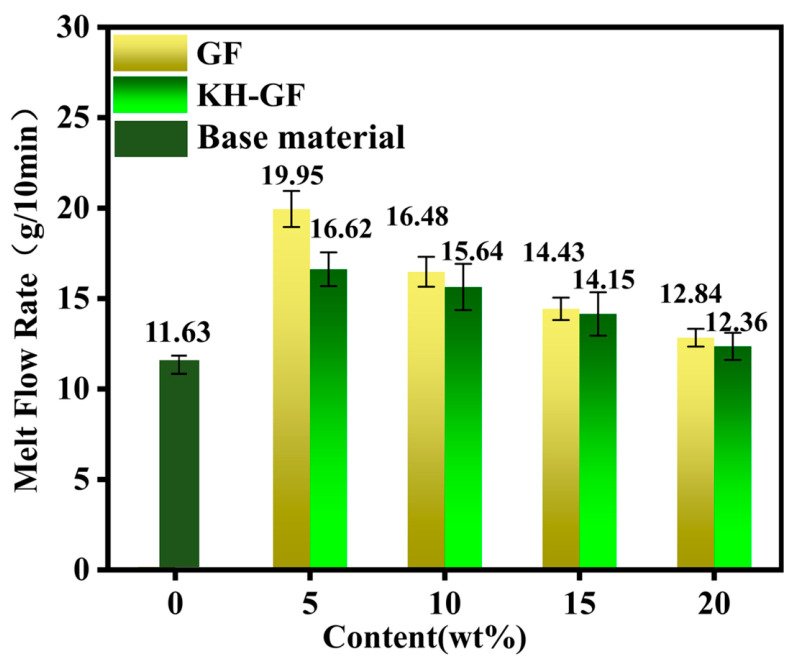
Melt flow rate (MFR) of modified composite materials with different additions of GF and KH-GF.

To investigate the rheological properties of PLA/PBS composite materials, the melt flow rate (MFR) of each component was determined. It is well known that the MFR reflects the flowability of a material during molding. A higher MFR indicates a lower molecular weight and better flowability, while a lower MFR indicates a higher molecular weight and better mechanical strength, toughness, hardness, and aging resistance. Figure 13 shows the MFR of GF and KH-GF-reinforced composite materials. From Figure 13, it can be observed that the composite material without GF has a relatively high MFR value, indicating good flowability and ease of processing. Conversely, its mechanical properties such as strength and toughness are relatively poor, which is consistent with the analysis of mechanical properties. However, as the GF content increases, both GF-reinforced and KH-GF-reinforced composite materials show a linear decrease in MFR values. This is mainly attributed to the increase in GF content, which improves the mechanical and thermal properties of the PLA/PBS composite material, resulting in decreased processing flowability. This trend is also reflected in the analysis of mechanical and thermal properties. However, the KH-GF composite material exhibits better mechanical properties. This phenomenon can also be observed in the injection-molded samples obtained during actual processing.

### 3.5. Morphological Characteristics

**Figure 14:**
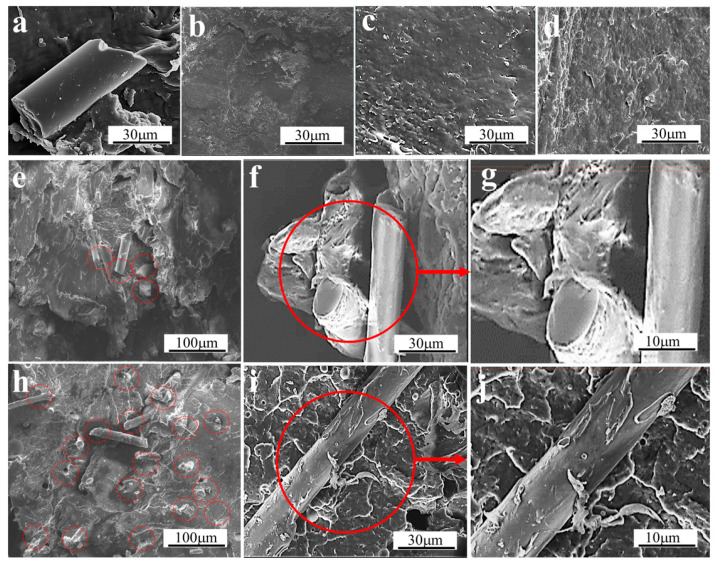
SEM images of the impact fracture surfaces and dispersion degree of glass fibers of modified composite materials with different additions of GF and KH-GF: (**a**) Morphology of GF, (**b**) Base material, (**c**) 10% GF, (**d**) 20% GF, (**e**) 10% GF dispersion, (**f**) 10% KH-GF, (**g**) Magnified morphology of 10% KH-GF, (**h**) 10% KH-GF dispersion, (**i**) 20% KH-GF, (**j**) Magnified morphology of 20% KH-GF.

Figure 14 shows the SEM images of the fracture surfaces of modified composite materials with different additions of GF and KH-GF and the dispersion of glass fibers. From Figure 14a, we can observe the appearance of a rod-like structure, which also indicates that GF can disperse in the PLA/PBS matrix, but as shown in Figure 14e, the degree of dispersion of GF in the matrix is not uniform. In addition, the surface of GF is smooth and exposed to the substrate. As a result, a good interface bond between GF and the PLA/PBS matrix cannot be formed, so that the impact performance of the composite does not achieve the expected effect. Figure 14b reveals a smooth fracture surface in the absence of GF, which is associated with the inherent brittleness of PLA itself. However, as the GF content increases, as observed in Figure 14c,d, the fracture surface becomes increasingly rough, accompanied by an increase in impact strength, indicating an improvement in mechanical properties with increasing GF content. The dispersion degree and fracture surface morphology of GF treated with the coupling agent are more satisfactory. It can be seen from Figure 14f–h that the dispersion degree of GF is relatively uniform. When the content of KH-GF is 10%, the PLA/PBS matrix exhibits a rough interface, and GF can effectively embed into the matrix. This may be due to the chemical functional groups Si-OH in the structure of the coupling agent combining with -OH and -COOH in the PLA and PBS structures, enhancing the interfacial strength between PLA and PBS, thereby exhibiting superior mechanical properties, especially a significant improvement in impact strength. Additionally, from Figure 14i,j, it can also be observed that with the increasing content of KH-GF, the GF surface becomes rougher and shows a stronger bond with the PLA/PBS matrix, resulting in an enhanced load-bearing capacity of the matrix and a substantial improvement in mechanical properties at the macroscopic level.

## 4. Conclusions

In this study, high-temperature resistant and tough PLA/PBS-based composite materials were prepared using the melt blending technique. The effects of different proportions of GF and KH-GF on the mechanical properties, crystallinity, and thermal performance of the PLA/PBS composite material were investigated. The results demonstrate that the addition of GF improves the interfacial bonding in the PLA/PBS composite material, resulting in a significant enhancement of its mechanical properties. When the KH-GF content reaches 20%, the tensile strength and modulus increase by 123% and 204%, respectively, while the flexural strength and modulus increase by 107% and 116%, respectively. The impact strength also shows an improvement of 189%. Furthermore, the modified GF with the coupling agent exhibits even better performance. When the KH-GF content is 5%, the cold crystallization temperature decreases, and the crystallinity increases from 34.72% to 41.28%. Additionally, as the GF content increases, the thermal stability of the composite material improves. According to the TGA data, when the KH-GF content is 20%, the final thermal decomposition temperature reaches 421 °C, and the Vicat softening temperature reaches 128.7 °C. In conclusion, the KH-GF-modified composite material shows superior mechanical properties and heat resistance compared to the GF-modified composite material, expanding the practical applications of PLA products and offering broader industrial prospects and commercial value.

## Figures and Tables

**Table 1:** Material Ratios (Parts per Hundred Resin, phr).

Sample	PLA	PBS	CaCO_3_	ADR4468	GF	KH-GF
Base material	70	30	3	3	/	/
GF5	70	30	3	3	5	/
GF10	70	30	3	3	10	/
GF15	70	30	3	3	15	/
GF20	70	30	3	3	20	/
KH-GF5	70	30	3	3	/	5
KH-GF10	70	30	3	3	/	10
KH-GF15	70	30	3	3	/	15
KH-GF20	70	30	3	3	/	20

**Table 2:** DSC data of modified composite materials with different additions of GF and KH-GF.

GF (wt%)	0	5		10		15		20	
Code	GF	GF	KH-GF	GF	KH-GF	GF	KH-GF	GF	KH-GF
T_C_ (°C)	79.53	74.02	71.78	76.34	71.24	77.01	71.76	77.64	74.51
∆H_C_ (J/g)	42.56	42.67	41.84	41.85	39.42	38.78	39.18	35.04	26.59
T_CC_ (°C)	103.26	101.33	98.64	100.18	97.52	101.85	99.52	102.03	99.58
∆H_CC_ (J/g)	1.94	4.25	4.03	4.75	4.14	2.98	4.23	2.42	2.61
T_m1_ (°C)	112.64	113.95	117.46	112.75	117.86	113.78	118.69	113.43	119.89
∆H_m1_ (J/g)	34.47	41.47	42.71	43.53	41.06	37.23	40.66	34.33	26.61
T_m2_ (°C)	144.12	144.53	141.84	144.81	143.28	145.23	147.58	145.61	148.24
∆H_m2_ (J/g)	1.88	2.09	3.44	1.93	3.78	1.98	1.87	1.16	1.37
χ (%)	34.72	39.72	41.28	41.39	45.29	36.75	40.38	34.09	39.53

**Table 3:** TGA data of modified composite materials with different additions of GF and KH-GF.

GF (wt%)	0	5		10		15		20	
Code	GF	GF	KH-GF	GF	KH-GF	GF	KH-GF	GF	KH-GF
T0	214	231	210	249	208	266	200	273	198
T5%	307	312	297	319	289	310	295	312	295
T10%	322	330	314	335	307	329	312	329	312
T50%	379	381	381	383	381	384	383	385	382
Td	418	421	422	422	421	423	422	421	420
Residue (%) at T ≥ 600 °C	4.56	5.53	10.93	9.25	12.17	14.75	13.63	15.82	15.47

## Data Availability

The data presented in this study are available in the article.
